# Intra-Operative Lymphatic Mapping and Sentinel Node Biopsy in Laryngeal Carcinoma: Preliminary Results

**Published:** 2015-07

**Authors:** Ehsan Khadivi, Maryam Daghighi, Kamran Khazaeni, Vahid Reza Dabbagh Kakhki, Leili Zarifmahmoudi, Ramin Sadeghi

**Affiliations:** 1 *Sinus and Surgical Endoscopic Research Center, Mashhad University of Medical Sciences, Mashhad, Iran.*; 2 *Nuclear Medicine Research Center, Mashhad University of Medical Sciences, Mashhad, Iran.*

**Keywords:** Laryngeal, Larynx, Radiotracer, Sentinel, SCC, Squamous Cell Carcinoma

## Abstract

**Introduction:**

Sentinel node mapping has been used for laryngeal carcinoma in several studies, with excellent results thus far. In the current study, we report our preliminary results on sentinel node mapping in laryngeal carcinoma using intra-operative peri-tumoral injection of a radiotracer.

**Materials and Methods:**

Patients with biopsy-proven squamous cell carcinoma of the larynx were included in the study. Two mCi/0.4 cc Tc-99m-phytate in four aliquots was injected on the day of surgery, after induction of anesthesia, in the sub-mucosal peri-tumoral location using a suspension laryngoscopy. After waiting for 10 minutes, a portable gamma probe was used to search for sentinel nodes. All patients underwent laryngectomy and modified radical bilateral neck dissection. All sentinel nodes and removed non-sentinel nodes were examined by hematoxylin and eosin (H&E) staining.

**Results:**

Ten patients with laryngeal carcinoma were included. At least one sentinel node could be detected in five patients (bilateral nodes in four patients). One patient had pathologically involved sentinel and non-sentinel nodes (no false-negative cases).

**Conclusion:**

Sentinel node mapping in laryngeal carcinoma is technically feasible using an intra-operative radiotracer injection. In order to evaluate the relationship of T-stage and the laterality of the tumor with accuracy, larger studies are needed.

## Introduction

Sentinel node mapping is the standard method of regional lymph node staging in breast cancer and melanoma (1,2). In this technique, the first draining lymph node(s) in the lymphatic drainage path of a tumor are identified (usually by radiotracers or blue dyes). These are called the sentinel node(s). The pathological condition of the sentinel nodes is considered a surrogate of the regional lymph node condition. Using this technique, the morbidity of the regional lymph node condition can be avoided in regional lymph node negative patients (3).

The sentinel node mapping concept has also been used with various degrees of success in gynecological, urological, and gastrointestinal tumors (4-7). For cutaneous and mucosal head and neck squamous cell carcinoma (SCC), sentinel node mapping has also been used with a high detection rate and sensitivity (8,9). Laryngeal SCC is a head and neck malignancy with different propensity for regional lymph node involvement. Location of the tumor (supraglottic vs. glottic/subglottic) and T-stage of the tumor are the variables related to the frequency of regional lymph node involvement in laryngeal SCC (10,11). Recommendations for regional lymph node dissection differ in the medical literature: in T3–T4 stage glottic cancer and in T2–T4 supra or subglottic tumors, elective neck dissection for clinically cervical node negative patients is recommended (11,12). Less invasive methods for regional lymph node staging in laryngeal carcinoma can reduce the complications of regional lymph node dissection as well as decreasing the surgery time. 

Sentinel node mapping has been used for laryngeal carcinoma in several studies, with excellent results thus far (13-15). In the current study, we report our preliminary results on sentinel node mapping in laryngeal carcinoma using an intra-operative peri-tumoral injection of the radiotracer.

## Materials and Methods

Ten patients with biopsy-proven SCC of the larynx were included in the study. Patients had no history of neck treatment, including radiotherapy or previous surgery. All patients were cN0 on neck examination and/or para-clinical imaging. Any of the following signs on computed tomography (CT) imaging were considered exclusion criteria: nodes larger than 10 mm, central necrosis, non-oval shape of nodes, perinodal tissue enhancement.

**Fig.1:**
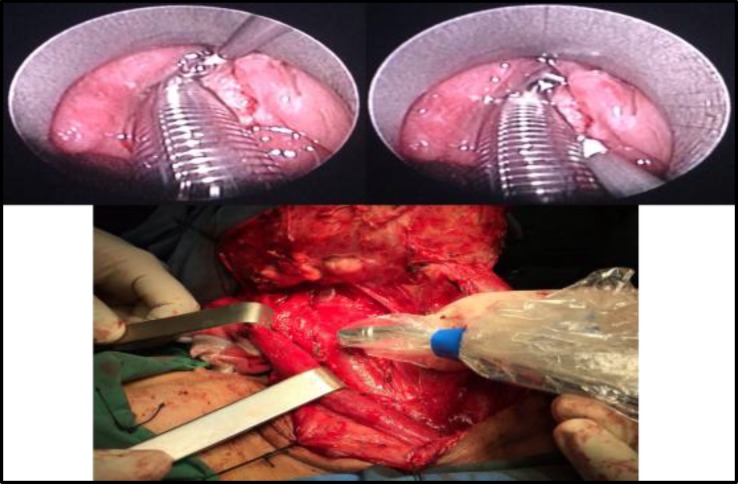
Intra-operative injection of the tracer and sentinel node mapping of a patient

On the day of surgery, after induction of anesthesia, 2 mCi/0.4 cc Tc-99m-phytate in four aliquots was injected in the sub-mucosal peri-tumoral location using a suspension laryngoscopy. After waiting for 10 minutes, sentinel nodes were searched for using a portable gamma probe (EURO PROBE, France). Any lymph node with more than a two-fold count rate as compared with the background was considered as sentinel node and harvested (Fig.1).

After excision of the sentinel nodes, the background of the neck area was checked for any residual activity. The location of the sentinel nodes was recorded according to their anatomical levels in the neck. All patients underwent laryngectomy and modified radical bilateral neck dissection. All sentinel nodes and removed non-sentinel nodes were examined by H&E staining. For sentinel nodes, step sectioning was also performed in order not to miss any small metastasis.

Detection rate was defined as the ratio of patients with at least one detected sentinel node to all recruited patients. The false-negative rate was defined as the ratio of patients with involved neck lymph nodes without involved sentinel node to all patients with involved neck nodes and at least a harvested sentinel node. This study was approved by the local ethical committee of Mashhad University of Medical Sciences under the approval number 920275. All patients gave written informed consent before inclusion into the trial.

## Results

Between May 2012 and Jan 2014, 10 patients with laryngeal SCC were included in the study. The mean age of the patients was 59.3 years (range: 48–69 years); nine patients were male and one was female (F/M: 1/9). All patients had T3 laryngeal tumors except for patient No. 5 who presented with T2 supra-glottic tumor. At least one sentinel node could be detected in five patients, which was bilateral in four patients (detection rate: 5/10=50%). One–to-four sentinel nodes could be identified in these patients. The median number of detected sentinel nodes in the patients was 2. Neck dissection yielded 6–51 lymph nodes. The median number of dissected lymph nodes was 24. One patient had pathologically involved sentinel and non-sentinel nodes (no false-negative cases). No other patient had lymph node involvement on pathological examination. Characteris- tics of the patients are shown in (Table. 1).

**Table 1:** Characteristics of the patients and results of the lymph node mapping

**Patient**	**Gender/age**	**Tumor location**	**T-stage**	**Number of sentinel nodes/number of harvested non-sentinel nodes**	**Location and laterality of sentinel nodes**	**Pathological sentinel node status**	**Pathological non-sentinel node status**
1	F/62	Epiglottis+ AEF+ FVC+ TVC+ Arytenoid+ Ant commissure	3	3/37	II ipsi; II contra	-	-
2	M/69	Epiglottis+ AEF+ FVC+ TVC+ Arytenoid+ Ant & pos commissure + pyriform sinus	3	0/27	N/A	-	-
3	M/48	FVC+ TVC+ Arytenoid + Ant commissure	3	1/15	III ipsi	-	-
4	M/66	AEF+ FVC+ TVC+ Arytenoid + Ant commissure + sub-glottis	3	0/13	N/A	-	-
5	M/48	Epiglottis + AEF + FVC	2	4/51	II, III ipsi; II, III contra	-	-
6	M/55	Epiglottis + AEF + FVC + TVC + Arytenoid + Ant & pos commissure +sub-glottis	3	2/25	II ipsi; II contra	One contralateral sentinel node positive	One contralateral non-sentinel positive (location station III)
7	M/50	TVC + Ant commissure + Arytenoid + sub-glottis	3	0/24	N/A	-	-
8	M/62	TVC + Ant commissure with extension to the base of the epiglottis	3	0/6	N/A	-	-
9	M/58	FVC + TVC + Arytenoid + Ant commissure + sub-glottis	3	0/20	N/A	-	-
10	M/75	TVC + Ant commissure with extension to the base of the epiglottis	3	2/42	II ipsi; III contra	-	-

AEF: Aryepiglottic fold; FVC: False vocal chord; TVC: True vocal chord

## Discussion

Sentinel node mapping is an important aspect of lymph node staging in several solid tumors of the head and neck, including melanoma, high risk SCC, and Merkel cell carcinoma (9,16,17). 

Several authors have previously reported their experience of sentinel node mapping in laryngeal carcinoma (13,15,18-31). The characteristics of these studies can be found (in Table. 2).

**Table 2:** Characteristics of previous studies of sentinel node mapping in laryngeal carcinoma

**Author/Year**	**Mapping material**	**Number of patients**	**Detection rate**	**Sensitivity**
Cheng 2010	Combined blue dye and radiotracer	40	82% by tracer, 66% by blue dye	83.3%
Fang 2001	Blue dye	23	95.6%	100%
Werner 2005	Radiotracer	11	100%	100%
Li 2009	Radiotracer	33	90.9%	93.3%
Werner 2002	Radiotracer	13	100%	100%
Chone 2008	Radiotracer	5	100%	N/A
Lawson 2010	Radiotracer	29	100%	100%
Lopez Molla 2006	Radiotracer	19	89.47%	50%
Prgomet 2008	Radiotracer	15	100%	100%
Hoft 2004	Radiotracer	12	91.6%	N/A
Yoshimoto 2012	Radiotracer	16	100%	100%
Tomifuji 2008	Radiotracer	19	100%	100%
Werner 2002	Radiotracer	14	100%	100%
Flach 2013	Radiotracer	19	68.4%	80%
Werner 2004	Radiotracer	31	100%	100%
Dunne 2004	radiotracer	12	100%	100%

Our preliminary results showed that sentinel node mapping using an intra-operative radiotracer injection is feasible in laryngeal carcinoma patients. We achieved a 50% detection rate and no false-negative cases. 

Tc-99m-phytate was used as the mapping material in this study as we have previously shown this to be a reliable tracer for sentinel node mapping (32). Intra-operative injection has also been used in several studies investigating different tumors (33) with excellent results as movement of the radiotracer around the lymphatic system is very fast and there is no need for a long wait after injection (34-37). As shown in Table 2, only two studies used blue dye as the mapping material and the remaining groups used only radiotracers for sentinel node mapping. We did not use blue dye and only relied on radiotracers for lymphatic mapping due to logistic problems and the risk of life-threatening complications by blue dyes, as previously reported in the literature (38).

The sentinel node detection rate in our study was sub-optimal (50%) as compared with the previous studies shown in Table 2. This may be due to several reasons. First, the learning curve effect could play an important role, as previous studies showed that surgeons gradually gain experience in sentinel node mapping with improvement in the detection rate (39). Second, the extension of tumors in our patient population was very wide and this could result in detection failure. As shown in Table 1, nine out of 10 included patients in our study had T3 tumors, while the other studies presented in Table 2 all recruited patients in the lower T-stages of T1 or T2. A higher T-stage increases the risk of lymphatic blockage, which invariably results in sentinel node detection failure (5,40,41). More patients are needed to further evaluate the relationship of T-stage and sentinel node detection failure in laryngeal carcinomas.

The larynx is a midline organ and can exhibit bilateral lymphatic drainage (20). This can have an important implication in sentinel node mapping. Midline tumors without bilateral detected sentinel nodes are a significant challenge for lymphatic mapping as this lymphatic pattern can result from lymph node involvement on one side with a higher risk of false-negative cases (42). Our results also showed that in four out of five patients with detected sentinel nodes, lymphatic drainage was bilateral. We need many more patients in order to evaluate the effect of laterality of lymphatic drainage on the accuracy of sentinel node mapping in laryngeal cancer. 

Another important aspect of sentinel node mapping is false-negative cases. No false-negative cases were revealed in this study, which is compatible with the results of the previous studies mentioned in Table 2. However, the number of patients with pathologically involved nodes remains low, and larger studies are needed in order to evaluate the false-negative rate more efficiently.

## Conclusion

Sentinel node mapping in laryngeal carcinoma is technically feasible using an intra-operative radiotracer injection. Larger studies are needed in order to evaluate the relationship of T-stage and the laterality of the tumor with accuracy of the technique. 
